# Transport and infrared photoresponse properties of InN nanorods/Si heterojunction

**DOI:** 10.1186/1556-276X-6-609

**Published:** 2011-11-28

**Authors:** Mahesh Kumar, Thirumaleshwara N Bhat, Mohana K Rajpalke, Basanta Roul, Ajit T Kalghatgi, S B Krupanidhi

**Affiliations:** 1 Materials Research Centre, Indian Institute of Science, Bangalore 560012, India; 2 Central Research Laboratory, Bharat Electronics, Bangalore 560013, India

## Abstract

The present work explores the electrical transport and infrared (IR) photoresponse properties of InN nanorods (NRs)/*n*-Si heterojunction grown by plasma-assisted molecular beam epitaxy. Single-crystalline wurtzite structure of InN NRs is verified by the X-ray diffraction and transmission electron microscopy. Raman measurements show that these wurtzite InN NRs have sharp peaks *E*_2_(high) at 490.2 cm^-1 ^and *A*_1_(LO) at 591 cm^-1^. The current transport mechanism of the NRs is limited by three types of mechanisms depending on applied bias voltages. The electrical transport properties of the device were studied in the range of 80 to 450 K. The faster rise and decay time indicate that the InN NRs/*n*-Si heterojunction is highly sensitive to IR light.

## Introduction

Semiconducting group-III nitrides have attracted a lot of attention in recent years because of, mainly, the large band gap (0.7 to 6.2 eV) that can be covered by the nitrides and their alloys. Their optical properties are highly suitable for novel optoelectronic and photonic applications. Compared to all other group-III nitrides, InN possesses the lowest effective mass, the highest mobility, narrow band gap *E*_g _of 0.7 to 0.9 eV, and the highest saturation velocity [1,2]. These properties make it an attractive material for applications in solar cells and in terahertz emitters and detectors [3-5]. InN/Si tandem cells have been proposed for high-efficiency solar cells [6]. InN nanostructures can also be used as sensor materials for various gases and liquids [7]. Good-quality InN layers are difficult to grow because of the low dissociation energy of InN and the lack of an appropriate substrates [8,9]. The above constraints lead to the formation of dislocations and strain in the grown epitaxial layers resulting in the degradation of the device performance. Grandal et al. [10] reported that defect- and strain-free InN nanostructures of very high crystal quality can be grown by molecular beam epitaxy on silicon substrates.

Due to the distinctive properties and potential applications of nanostructures, various kinds of InN nanostructures have been grown such as nanowires (NWs), nanotubes, and nanorods (NRs) by plasma-assisted molecular beam epitaxy (PAMBE) and metalorganic vapor phase epitaxy [11,12]. There are several reports on the growth of InN NWs or NRs on Si substrates [13,14] and few reports on electrical transport [15,16] but no report on infrared (IR) on/off characteristics of InN nanorods/Si heterojunction. Since silicon is a low-cost and the most sought semiconductor material, it is very important to understand the temperature-dependent transport and IR photoresponse mechanism of InN NRs/Si heterostructure prior to their adoption in the fabrication of optoelectronic device. In the present study, catalyst-free InN NRs were grown on Si substrates by PAMBE and studied the temperature-dependent transport and IR photoresponse mechanism of InN NRs/Si heterostructures.

## Experimental details

The InN NRs were grown on *n*-Si (1 1 1) substrates by PAMBE system. The substrates were chemically cleaned followed by dipping in 5% HF to remove the surface oxide and thermally cleaned at 900°C for an hour in ultra-high vacuum. The substrates were exposed to the Indium (In) molecular beam at 350°C for 60 s (approximately two monolayers). Further, the substrate temperature was increased to 500°C to fabricate the NRs. The duration of NR growth was kept for 2 h. The general set of growth conditions includes indium beam equivalent pressure, nitrogen flow rate, and rf-plasma power, which were kept at 4.6 × 10^-8 ^mbar, 1 SCCM, and 400 W, respectively. The morphological and structural evaluation of the as-grown NRs was carried out by the field emission scanning electron microscopy (FESEM), X-ray diffraction (XRD), and transmission electron microscopy (TEM). Further, the crystalline quality and lattice structure of the InN NRs were characterized by micro-Raman spectroscopy using a 514-nm line of the Ar^+ ^ion laser at room temperature. The aluminum circular contacts of diameter 400 μm were fabricated by thermal evaporation using a physical mask. The adequate ohmic nature of the contacts to InN and Si was verified. The device transport characteristics were studied at various temperatures using the probe station attached with the KEITHLEY-236 source measure unit (Bell Electronics, Kent, WA, USA), and IR photoresponse characteristics were studied under IR source with 1, 500-nm-long pass filter.

## Results and discussion

**Figure 1:**
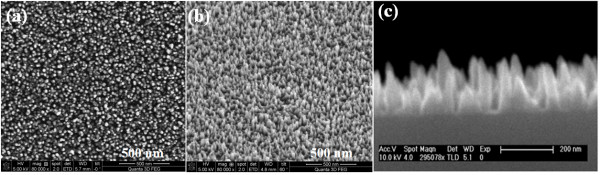
**FESEM images**. (**a**) Top view, (**b**) 60° tilted view, and (**c**) cross-section view of InN NRs.

**Figure 2:**
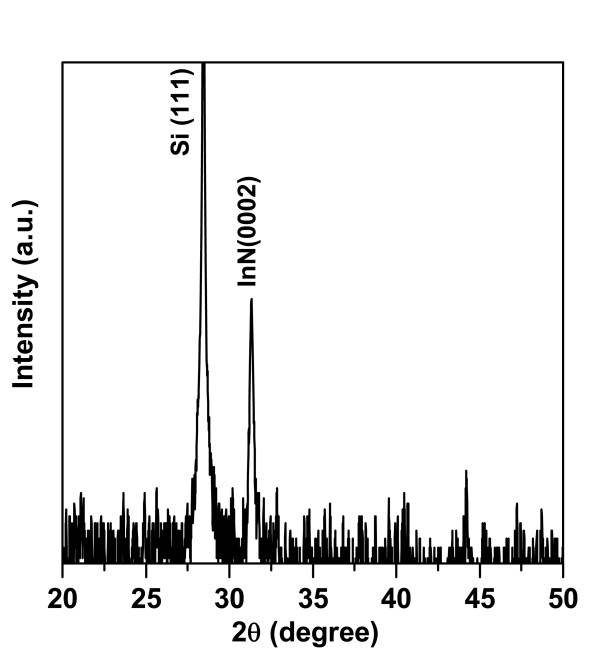
**X-ray diffraction measurement of the InN NRs on *n*-Si**.

**Figure 3:**
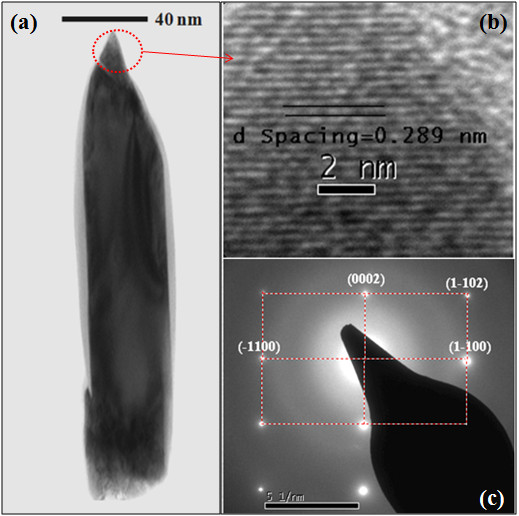
**TEM, HRTEM, and SAED images.(a)** TEM image, **(b)** HRTEM image of an InN NR taken on the tip of nanorod and **(c) **SAED pattern of an InN NR.

**Figure 4:**
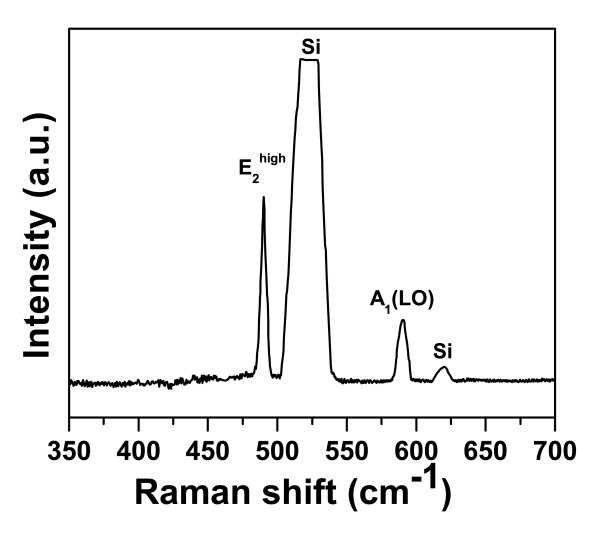
**Raman spectrum of InN NRs, showing the *E*_2_(high) and *A*_1_(LO) phonon modes**.

Figure 1a, b, c FESEM images show top view, 60° tilted view, and the cross-section view of InN NRs, respectively. It is seen in the figure that the as-grown nanorods are uniformly grown over the entire substrate. The average length and diameter of these rods are found to be approximately 200 nm and approximately 40 nm, respectively. The structural characteristics of the as-grown InN NRs were evaluated by XRD. Figure 2 shows 2*θ*-*ω *scan of the InN NRs grown on Si (1 1 1) substrates. The peak at 2*θ *= 31.34° is assigned to the (0 0 0 2) planes of the InN, indicating that the InN NRs to be highly oriented along the [0 0 0 1] direction of the wurtzite structures of InN. Figure 3a, b, c represents typical transmission electron micrographs, high-resolution TEM (HRTEM), and selected area electron diffraction (SAED) images of single InN NR, respectively. The HRTEM was taken on the tip of the NR, and the interplanar spacing, as observed from the fringe pattern of the HRTEM image, is 0.289 nm, which corresponds to the (0 0 0 2) lattice spacing of InN [17]. The SAED pattern shows clearly visible bright spots which represents that each NR is single crystalline. These results clearly demonstrate that the as-grown NRs are fairly single crystalline and are crystallized hexagonally along the [0 0 0 1] direction with uniform geometry. The crystalline quality and the lattice structure of the InN NRs were further investigated by Raman spectroscopy. A typical room temperature Raman spectrum is shown in Figure 4. Two active phonon modes were observed at around 490.2 and 591 cm^-1^, corresponding to the *E*_2_(high) and *A*_1_(LO) modes of InN, respectively. These two modes agree well with the previous reports on InN [17,18], and these observations are also in good agreement with HRTEM images, which show that the NRs are crystallized hexagonally along the [0 0 0 1] direction. The strain-free Raman frequency of the *E*_2_(high) mode has been reported to be 490 cm^-1 ^for wurtzite InN [19]. The *E*_2_(high) mode frequency observed in our experiment is very close to this reported value, within an instrumental error, which indicates that the strain of the InN NRs is fully relaxed.

**Figure 5:**
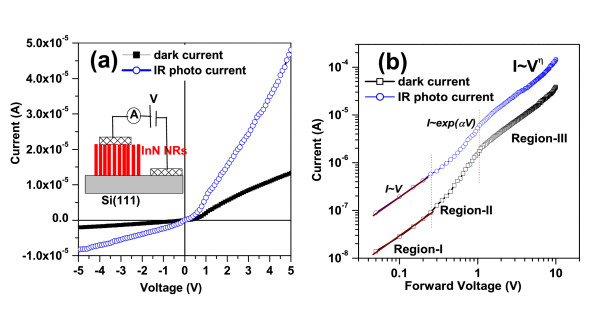
**Dark and IR photocurrent versus voltage plots, and log-log plots of the current-voltage**. (**a**) Dark and IR photocurrent versus voltage plots of the InN NRs/*n*-Si heterojunction diode. The inset shows the schematic diagram of diode. (**b**) Log-log plots of the current-voltage under forward bias of InN NRs/*n*-Si heterojunction measured at room temperature. The *I*-*V *plot is divided into three different regions.

**Figure 6:**
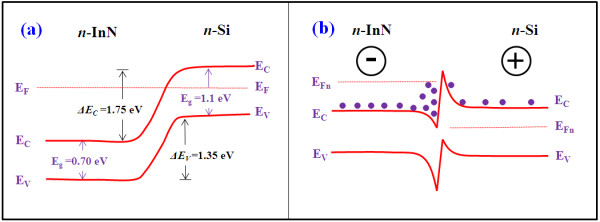
**The energy band diagrams of InN/Si heterojunctions**. Under (**a**) zero bias and (**b**) reverse bias.

The *I*-*V *characteristic of the InN NRs/Si heterojunction are measured at room temperature, and a schematic diagram of the heterojunction diode is shown in the inset of Figure 5a. The carrier densities of *n*-Si were found to be 2 × 10^17 ^cm^-3^, as measured by Hall measurements, and the InN NRs were unintentionally doped which show an *n*^+^-type conductivity [20]. The diode shows poor rectifying behavior with an on/off ratio (*I*_F_/*I*_R_) 6.1 at 3 V and 6.8 at 5 V. The leakage current is -1.4 × 10^-6 ^A at -3 V. The on/off ratio decreased to 2.9 at 3 V and 3.6 at 5 V under the IR source illumination. Additionally, the leakage current is increased to -5.5 × 10^-6 ^A at -3 V, about three times higher than the dark leakage current. To understand the rectifying behavior observed in these *n*-*n *InN NRs/Si heterojunctions, one first notes that there exists an offset between the conduction bands of InN and Si. The energy band diagrams of InN/Si heterojunctions under zero bias and forward bias that are derived from Anderson model are shown in Figure 6a, b, respectively. The electron affinities of InN and Si were taken as 5.8 and 4.05 eV, and the bandgaps were taken as 0.7 and 1.1 eV, respectively. This type of behavior is also observed in *n*-*n *GaN/SiC and *n*-*n*^+ ^GaN/GaAs heterostructures, which is attributed to Fermi-level pinning by interface defects [21,22]. The current-voltage log-log scale plot for the InN NRs is shown in Figure 5b. The log-log plot can be divided into three different regions depending on the applied voltage. In the first region (*V *< 0.4 V) at a very low forward voltage, a linear dependence of the current on the voltage (*I *≈ *V*) is observed which follows a transport mechanism obeying the Ohm's law. In the second region (0.4 V <*V *< 1.2 V), the current is increased exponentially with a relation *I *≈ exp(*αV*) which is usually observed due to a recombination tunneling mechanism [23], where *α *is constant and *V *is applied forward bias. In the third region (1.2 V <*V <*10 V) at a moderately higher junction voltage, the *I*-*V *characteristics follow a power law *I *≈ *V^η^*, where *η *= 1.6 for dark current and 1.8 for IR photocurrent. In the third region, the current conduction is attributed to the space-charge-limited current.

**Figure 7:**
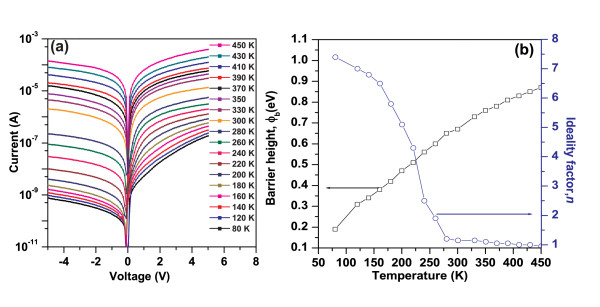
**Current versus voltage plots and variation of barrier heights and ideality factor**. (**a**) Current versus voltage plots of the InN NRs/*n*-Si diode at different temperatures. (**b**) Variation of barrier heights and ideality factor with temperature.

Figure 7a shows the temperature-dependent *I*-*V *characteristics of the junction in the temperature range of 80-450 K. An excellent rectifying behavior was observed at lower temperatures with an on/off ratio of 240 at 5 V and at 80 K. The deterioration observed in the rectifying nature at high temperature may be due to thermally generated carrier tunneling. Firstly, it is very clear from the *I*-*V*-*T *curve that, at fixed bias, the forward current increases with increasing temperature, which indicates that the current is induced by the thermionic emission (TE). The values of barrier height (*ϕ*_b_) and the ideality factor (*η*) for the junction were calculated as a function of measuring temperature by fitting a line in the linear region of the forward *I*-*V *curves using the TE model:

$$I=I_{\text{s}}\left[\exp\left(\frac{qV}{\eta kT}\right)-1\right]\;\text{where}\;I_{\text{s}}=AA^{*}T^{2}xp\left(-\frac{\phi_{\text{b}}}{kT}\right)$$

From Figure 7b, it can be seen that the barrier height (*ϕ*_b_) and the ideality factor (*η*) are dependent on the temperature and are attributed to the inhomogeneity at the interface [24]. The large observed ideality factor suggests the presence of surface or interface states, indicating that the junction is far from being ideal [25,26]. The large mismatch in the lattice parameters of InN and Si shows that there is generally a high density of interfacial states between two materials. The lattice mismatch produces a dislocation field at the junction interface that can attract a space charge and/or act as a recombination center, resulting in large ideality factor. Breitenstein et al. [27] introduced a model to describe ideality factors *n *> 2, which is based on coupled defects and donor acceptor pair recombination, both giving rise to an increased recombination current. It is stated that for a high density of defect states, hopping conduction in the defect volume may govern the reverse conductivity of the devices.

**Figure 8:**
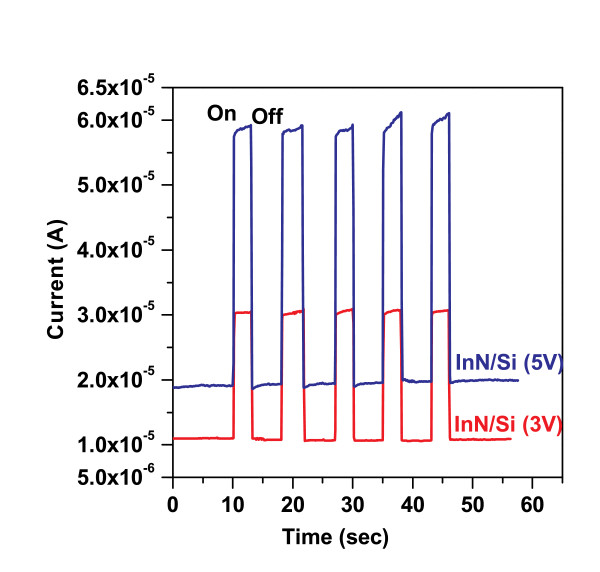
**A logarithmic plot of the transient photocurrent of InN NRs/*n*-Si heterojunction**. Measured at 3 and 5 V bias conditions.

The NRs show features at 0.77 eV in photoluminescence at 10 K ascribed to band edge emission. Photocurrent transient measurement of the InN NRs/*n*-Si heterojunction (Figure 8) shows that the heterojunction can be turned "on" and "off" by switching the illumination of IR source (*λ *< 1, 500 nm blocked by long pass filter) at 3 and 5 V bias with an on/off ratio of 2.9 and 3.5. The time constants for the rise *τ*_r _and decay *τ*_d _of the photocurrents are estimated to be less than approximately 150 ms from following equations: *I *= *I*_o_(1-exp(-*t*/*τ*_r_) and *I *= *I*_o_(1-exp(-*t*/*τ*_d_) for rise and decay, respectively. This rapid rise and decay indicate that the InN NRs/*n*-Si heterojunction is very sensitive to the IR light.

## Conclusions

InN NRs/*n*-Si heterojunction was grown by PAMBE. Single-crystalline wurtzite structure of InN NRs is verified by the X-ray diffraction and HRTEM. Raman spectrum reveals two clear peaks, which correspond to the *E*_2_(high) and *A*_1_(LO) modes of wurtzite InN, respectively. The current transport mechanism of the NRs/Si heterojunctions were limited by three types of mechanisms depending on applied bias voltages. The observed higher value of ideality factor is probably due to the presence of defect states in InN NRs. The rapid rise and decay of infrared on/off characteristics of InN nanorods/Si heterojunction indicate that the device is highly sensitive to the IR light. The InN NRs/Si heterojunction device can be used for IR detectors.

## Competing interests

The authors declare that they have no competing interests.

## Authors' contributions

The work presented here was carried out in collaboration between all authors. MK, AKT, and SBK defined the research theme. MK, TNB, BR, and MKR designed methods and experiments and also coordinated the present study. MK carried out the laboratory experiments, interpreted the results, and wrote the paper. All authors have contributed to, seen, read, and approved the manuscript.
